# Administration of vitamin E and C enhances immunological and biochemical responses against toxicity of silver nanoparticles in grass carp (*Ctenopharyngodon idella*)

**DOI:** 10.1371/journal.pone.0284285

**Published:** 2023-04-27

**Authors:** Khalid Javed Iqbal, Hamid Majeed, Khalid Jamshed Iqbal, Muhammad Asghar, Hamda Azmat, Mahroze Fatima, Noor Khan, Irfan Baboo, Amna Tehseen, Wazir Ali, Usama Saeed, Ayesha Khizar, Amber Fatima, Sobia Nisa, Simon John Davies

**Affiliations:** 1 Department of Zoology, The Islamia University of Bahawalpur, Bahawalpur, Pakistan; 2 Department of Food Science and Technology, Cholistan University of Veterinary and Animal Sciences, Bahawalpur, Pakistan; 3 Department of Chemistry, Khawaja Fareed University of Engineering and Information Technology, Rahim Yar Khan, Pakistan; 4 Department of Fisheries and Aquaculture, University of Veterinary and Animal Sciences, Lahore, Pakistan; 5 Institute of Zoology, University of the Punjab, Lahore, Pakistan; 6 Department of Zoology, Cholistan University of Veterinary and Animal Sciences, Bahawalpur, Pakistan; 7 Department of Biology, University of Copenhagen, Copenhagen, Denmark; 8 College of Science and Engineering, National University of Ireland, Galway, Ireland, United Kingdom; Patuakhali Science and Technology University, BANGLADESH

## Abstract

The aim of the current study was to evaluate the toxic effect of silver nanoparticles (Ag-NPs) on biochemical biomarkers, immune responses, and the curative potential effects of vitamin C and E on grass carp. Fish (n = 420) with an average initial body weight of 8.045 ± 0.13 g were shifted to glass aquaria (36 x 18 x 18 inches, filled with 160-L tap water) in triplicates. Aquaria were randomly designated as A, B, C, D with alone Ag-NPs (Control (0), 0.25, 0.50, 0.75 mg/L) and E, F, G with Ag-NPs + Vit. C + Vit. E (0.25+0.25+0.25, 0.50+0.50+0.50, 0.75+0.75+0.75 mg/L). NPs particles were administrated viz, oral and intravenous routes for 7 days. The results indicated that both routes had non-significant effect, but levels of Ag-NPs had significant effect. Treatments C, D and G showed significant decrease in levels of RBC, HGB and HCT except for WBC and NEUT levels, which significantly increased. ALT, ALP, AST, urea, and creatinine showed significant increase in activity in the C, D, and G groups. CAT, SOD decreased significantly in all Ag-NPs alone groups, while significantly increased with vitamin E and C. LYZ, TP, ALB, GLB showed significant low activity in the B, C, and D groups while significantly high activity in the E, F, and G groups. Cortisol, glucose and triglycerides showed significant increase in the B, C, and D groups, while E, F, and G groups showed significant low levels of triglycerides, COR, and GLU. Cholesterol level was same across all treatment groups. In conclusion, vitamin E and C as powerful antioxidants protect the fish against Ag-NPs except high dose level of 0.75mg/L, while 0.25mg/L of Ag-NPs was presumably safe for *C*. *idella*.

## 1. Introduction

All instances of nano-technology include understanding, manipulating, and applying materials at the Nano scale (1 to 100 nm). Nano-particles have distinct advantages over bulk materials, making them valuable in a broad variety of industries and consumer goods [1]. Silver nanoparticles (Ag-NPs) production and usage have increased considerably in recent years, accounting for more than 50% of global nanomaterial products in 2015, and this proportion is projected to climb by nearly 13% by 2024. The main reasons for this are Ag-NPs antimicrobial, optical, electrical, and magnetic properties. Because of their small size, nanoparticles are extensively used in a broad range of goods such as make-up and textiles all over the globe [2, 3]. Concerns have been raised regarding the release of Ag-NPs into the environment due to the high concentrations found in sewage sludge and effluent (in Europe and the US) of 1.3–4.4 mg/kg, as well as 9 μg/L (in Europe) and 10^−1^–10^−4^ μg/L (Europe, USA, Netherlands, European Union) in surface waters. An emerging field of research, aquatic nano-toxicology, has piqued attention. Aquatic animals are exposed to nanoparticles via a variety of sources, including food, water, and sediment [3].

According to research, Ag-NPs hurt various fish and mammals [3–5]. Pharmaceutical companies measure animal sensitivity and survival potential using LC50 tests [6]. Carps were exposed to a fatal dose of Ag-NPs for 96 hours [7]. Fish exposed to varying sub-lethal concentrations of Ag-NP LC50 (96h) showed changes in plasma biochemical concentrations (such as electrolytes and proteins), oxidative stress/cell damage markers (such as glucose and cortisol), lactate dehydrogenase, aspartate aminotransferases, and alanine aminotransferases, antioxidant enzymes, and lipid peroxidation [4, 8]. Recent research found that chemically manufactured nanoparticles are 10 times more hazardous than green ones [9]. There are two techniques for NP synthesis [10]. Sputtering, etching, mechanical milling, and electro-explosion are used in one approach, while physical, chemical, and biological methods are used in the other [11]. Although traditionally produced NPs are pure, they are not cost-effective and may produce dangerous byproducts that have negative effects. These procedures need stabilizing and capping agents [12]. Plant extracts serve as the reducing medium in the green synthesis process, while phytochemicals serve as the capping agent, biocatalyst, and naturally occurring NP stabilizer [13]. High energy, high temperature, high pressure, costly equipment, and dangerous chemicals are not necessary in green synthesis of NPs [14]. Consequently, green synthesis of NPs is less expensive, non-toxic, and environmentally beneficial than pricy and dangerous methods [15, 16]. Very little is known about the toxicity of green produced NPs to fish. According to a prior research, common carp respond to exposure to waterborne NPs [17].

The plant *Azadirachta* (A.) *indica*, sometimes referred to as "neem," leaves are employed in current research for the production of Ag-NPs. The anti-ulcer, anti-tumor, anti-bacterial, analgesic, anti-yeast, anti-inflammatory, anti-fungal, anti-hyperglycemic, anthelminthic, and anti-malarial properties of *A*. *indica* leaves have been noted. Its leaves have been used to treat chicken pox, malaria, and neuromuscular problems [18]. Knowing what physiological changes occur when an important freshwater aquaculture species is exposed to green synthesize Ag-NPs enables us to establish a safe exposure threshold [19]. Hematology helps assess nanoparticle toxicity for aquatic animals [2]. Serum and hematological indicators are used to determine fish health [20]. They can assess the general status of cells, tissues, and organs as well as their pollutant-damaged targets [21]. Scientists acknowledge hematological indices and serum biochemical as significant stress indicators and critical biomarkers for fish health status [8, 21].

Supplements in fish diet may boost immune responses and health, raise growth rate, and protect fish from toxic toxins, influencing their physiology [22]. Most teleost fish lack the vitamin C-making enzyme L-gulonolactone oxidase [23]. This enzyme makes vitamin C. Vitamin C, is present in many foods and nutritional supplements [24]. It removes damaging free radicals to prevent lipid membrane instability [25]. It decreases environmental health risks [26, 27].

Tocopherol (vitamin E), a non-enzymatic antioxidant, may help the body deal with oxidative stress by decreasing circulating lipid peroxides [28]. The effects of vitamin E as a molecule are yet unknown, despite the growing body of research on its ability to inhibit oxidative stress [29]. Furthermore, the effects of Ag-NPs, vitamin C, and vitamin E on fish are completely unknown. Grass carp is an important cultivated profitable fish and a global distribution. Grass carp global production is about 4.6 million tons/year, accounting for 15.6% of global freshwater aquaculture production in 2011 [30]. It has very good taste and fast growth rate. In Pakistan it was introduced for the first time in 1994 from China [31].There is no information about the impact of green synthesized Ag-NPs on the aforementioned indices like hematological, serum and antioxidant parameters in Grass carp (*C*. *idella*), or whether or not vitamins C and E may mitigate the damage.

## 2. Materials and methods

### 2.1 Ethical approval, experimental fish, design and conditions

All protocol and procedure was approved by guidelines for care and use of laboratory animals committee of The Islamia University Bahawalpur, Pakistan (No. Dr/792, 21-09-2022). For this study, 420 *C*. *idella* were obtained from the Bahawalpur fish hatchery, Government of Punjab, Pakistan. These fish had an average weight of 8.045±0.13g. The fish were delivered safely to the Department of Zoology at The Islamia University in Bahawalpur, Pakistan, within air-filled plastic bags. The fish were acclimated in laboratory aquariums for two weeks to reduce their stress levels. During the adjustment and adaptation phase, no deaths were reported. The fish were given commercial feed that contained 32% CP @ 3% body weight ratio twice a day. Particles of Ag-NPs were given orally and intravenously to *C*. *idella*. Three replicates of 42 glass aquariums (36 x 18 x 18 inches, filled with 160 liters of tap water) were set up, and fish were randomly distributed among them with the following designations: A (Control, Ag-NPs, 0.00 mg/L), B (Ag-NPs, 0.25 mg/L), C (Ag-NPs, 0.50 mg/L), D (Ag-NPs, 0.75 mg/L), E (Ag-NPs + Vit. C + Vit. E, 0.25+0.25+0.25 mg/L), F (Ag-NPs + Vit. C + Vit. E, 0.50+0.50+0.50 mg/L) and G (Ag-NPs + Vit. C + Vit. E, 0.75+0.75+0.75 mg/L). Both intravenous and oral aquaria were set up at the same time. The duration of the trial was 7 days. Through the gastric tube attached to the syringe, the medication was orally ingested and intravenously injected into the caudal vein. Dissolved oxygen levels were kept at 7.1±0.2 mg/L, water temperature was kept at 28.5±0.3°C, and the pH was kept at 7.4±0.1 throughout the duration of the experiment. Fish were subjected to a 12-hour photoperiod in a controlled laboratory setting.

### 2.2 Synthesis of silvers nano particles

Using *Azadirachta indica* (Neem) leaf extract as a reducing agent, silver nanoparticles were synthesized biologically. Twenty grams of dried *A*. *indica* powder were mixed into one hundred milliliters of distilled water to make the extract. After 45 minutes at 40°C on a hot plate, the solution was cooled, filtered using Whatman filter paper, and placed in the fridge for later use. The silver nitrate solution was made up in a Sigma-Aldrich Chemical Co. reagent container of 200 ml capacity (1 mM). When the solution’s colour shifted from pale to colloidal brown, indicating the creation of nanoparticles, 5 ml of plant extract was added and agitated on a hot plate [32]. For 15 minutes, we centrifuged the solution at 6000 rpm. When 24 hours at 37°C in a beaker, the pellets were dry and ready to use after the supernatant was drained. By using the XRD method, the size of the Ag-NPs was determined to be 21 nm, and they were also detected using UV-Spectroscopy. LC50 was determined during preliminary experiment by following, 100 individuals of fish were placed in 10 glass aquaria (10 fish each) to determine 96Hr LC50 of grass carp. Stock solution of Ag-NPs was prepared and a total of ten concentrations were set in geometric series until the onset of LC50. Mortality of fish was noted and subject to probate analysis for final value of LC50. Then, green synthesized silver nanoparticles (Ag-NPs) were administered orally and intravenously in *C*. *idella*.

### 2.3 Blood and serum collection

At the end of the first week of the experiment, five fish were taken at random from each tank and given MS222 (150mg/L) to put them to sleep. Using a syringe, blood was drawn from the caudal vein. Blood was kept in EDTA blood vacutainers, which prevent the blood from clotting, for hematological purposes. Additionally, gel vacutainers were used to hold blood for serum biochemistry. After letting the blood clot, it was centrifuged at 3000 rpm for 5 minutes at 4°C to remove the serum, which was then frozen at -80°C until further analysis was performed.

### 2.4 Hematology parameters

An automated hematological analyzer was used in order to conduct the test on the blood (Sysmex KX-21N). Red blood cell count (RBC), white blood cell count (WBC), hemoglobin (Hb), hematocrit (HCT), mean corpuscular volume (MCV), mean corpuscular hemoglobin (MCH), and mean corpuscular hemoglobin concentration (MCHC) were the parameters that were examined in fish. Other parameters included lymphocytes (LYM), monocytes (MONO), and neutrophils (NEUT).

### 2.5 Serum biochemistry and immunology

Serum tests for urea, creatinine, alkaline phosphatase (ALP), aspartate transaminase (AST), and alanine transaminase (ALT), total protein (TP), glucose (GLU), cholesterol (CHO), albumin (ALB), and triglycerides were run by an automated biochemical analyzer (Micro Lab-300) (Tri). The ratio of total serum protein to albumin is used to calculate serum globulin (GLB) concentration [33].

### 2.6 Antioxidant and lysozyme activity

According to [34] approach, catalase (CAT) activity was measured by its capacity to lower the concentration of -H_2_O_2_ at 20nm. While photo reduction inhibition of nitro blue tetrazole (NBT) according to the technique of [35] was used to test superoxide dismutase (SOD) activity. Lysozyme (LYZ) activity was evaluated using [36] methodology.

### 2.7 Statistical analysis

Effects of silver nano particles on hematology, serum biochemistry, immunology, and antioxidant and lysozyme activity were evaluated by using factorial ANOVA. PROC GLM was used in SAS software (version 9.1). A 2 × 7 factorial arrangement of treatments were applied considering rout of administration and silver nano particle levels as main effects and their interaction were tested, too. For the comparison of significant treatment means Duncan’s Multiple Range test was applied considering P<0.05. Graphs were created by using GpraphPad Prism software. Following mathematical model was applied:

$$Y_{ijk}=\mu +\alpha_{i}+\beta_{j}+{\left(\alpha \times \beta \right)}_{ij}+\epsilon_{ijk}$$

Where,

Y_ijk_ = Observation of dependent variable recorded on i^th^ and j^th^ treatment groups

μ = Population mean

α_i_ = Effect of i^th^ treatment group (rout of administration; i = 1, 2)

β_j_ = Effect of j^th^ treatment group (silver nano particle levels; j = 1, 2, 3, 4, 5, 6, 7)

(α × β)_ij_ = Overall interaction effect

ϵ_ijk_ = Residual effect associated with i^th^ and j^th^ treatment group, NID ~ 0, σ^2^

## 3. Results

Hematological parameters like RBC, WBC, HB, HCT, MCV, MCHC, LYM, MONO, and NEUT showed non-significant (P>0.05) differences with respect to interaction of dose level and dose route (oral or intravenous routes) of Ag-NPs (Table 2). Also, the mean effect of Ag-NPs on both dose routes, either oral or intravenous showed non-significant (P>0.05) difference (Table 1). However silver nanoparticles alone or with supplementation of vitamin E and C showed significant (P<0.05) mean effect on hematology with respect to different dose levels (Table 1). RBC, HGB, and HCT levels decreased significantly (P<0.05) at Ag-NPs levels of 0.50 and 0.75mg/L as compared to control and 0.25mg/L Ag-NPs. Similarly, Ag-NPs concentration of 0.75mg/L along with the supplementation of vitamin E and C showed significant (P<0.05) decrease in RBC, HGB and HCT with respect to other vitamin supplemented groups and control. WBC and NEUT showed the opposite trend; at 0.50 and 0.75mg/L NPs alone and 0.75mg/L NPs + vitamin E and C administration, their levels increased significantly (P<0.05) as compared to control, but at 0.25mg/L NPs dose, there was a non-significant (P>0.05) difference with control. While at 0.25 and 0.50mg/L NPs + vitamin E and C ingestion, levels of WBC and NEUT decreased significantly (P<0.05) as compared to others and there was a non-significant (P>0.05) difference with control. While MCV, MCH, MCHC, LYM and MONO showed non-significant (P>0.05) effect of Ag-NPs alone or with vitamin E and C (Table 1).

**Table 1:** Mean effect of Ag-NPs alone and Ag-NPs + V.E + V.C with respect to dose route and dose levels on hematology of *C*. *idella*.

Dose Route	Parameters									
	RBC^1^ (10^6^/μl)	WBC^2^ (10^3^/μl)	HGB^3^ (g/dl)	HCT^4^ (%)	MCV^5^ (nm^3^)	MCH^6^ (pg/cell)	MCHC^7^ (g/dl)	LYM^8^ (%)	MONO^9^ (%)	NEUT^10^ (%)
**Oral**	1.93±0.20	185.92±19.54	8.54±0.84	31.85±5.02	167.10±1.63	168.01±1.21	31.40±0.78	82.79±1.26	2.40±0.08	24.53±4.18
**Intravenous**	1.92±0.20	185.97±18.86	8.51±0.87	31.73±4.78	167.24±1.50	167.86±1.23	30.60±0.67	82.47±1.17	2.39±0.07	24.53±4.32
**P-value**	0.89	0.93	0.24	0.57	0.77	0.71	0.07	0.42	0.50	.99
**Dose levels**										
**A**	2.09±0.01^d^	171.74±1.27^a^	9.15±0.02^d^	35.67±0.64^d^	168.18±1.71	168.18±1.71	31.11±0.69	82.48±1.18	2.44±0.09	21.07±1.00^a^
**B**	2.08±0.01^cd^	172.70±1.13^a^	9.17±0.02^d^	35.27±0.77^d^	168.13±1.68	168.46±1.42	30.70±0.44	82.46±1.24	2.37±0.06	21.39±1.23^a^
**C**	1.94±0.1^cd^	185.98±2.57^b^	8.29±0.01^c^	29.97±0.54^c^	166.28±0.73	167.61±1.07	31.17±0.65	82.93±1.54	2.35±0.01	26.63±0.85^b^
**D**	1.58±0.02^a^	223.31±2.80^d^	6.80±0.16^a^	23.34±0.63^a^	166.77±1.49	167.94±1.11	30.68±1.11	81.71±0.45	2.40±0.05	31.90±1.07^d^
**E**	1.93±0.26^c^	172.51±1.32^a^	9.15±0.02^d^	35.58±0.89^d^	166.41±0.99	167.41±0.55	30.84±0.86	83.17±1.28	2.44±0.11	21.26±0.84^a^
**F**	2.08±0.01^cd^	172.40±1.57^a^	9.15±0.03^d^	35.91±0.75^d^	167.26±1.86	168.43±1.29	30.53±0.57	83.12±1.02	2.43±0.09	21.26±0.61^a^
**G**	1.77±0.01^b^	202.94±2.12^c^	7.99±0.03^b^	26.81±0.34^b^	167.17±1.60	167.50±1.14	30.73±0.95	82.55±1.41	2.34±0.09	28.23±0.78^c^
**P-value**	0.000	0.000	0.000	0.000	0.20	0.67	0.56	0.49	0.13	0.000

Data are presented as mean ± SD. Data were analyzed through two-way ANOVA besides Duncan comparisons. Data with different superscript letters (a, b, c, d, e and f) in the same column mean significant differences among experimental groups (P<0.05). ^1^Red blood cell, ^2^White blood cell, ^3^Hemoglobin, ^4^Hematocrit, ^5^Mean cell volume, ^6^Mean cell hemoglobin, ^7^Mean corpuscular hemoglobin concentration, ^8^Lymphocytes, ^9^Monocytes, and ^10^Neutrophils

**Fig 1:**
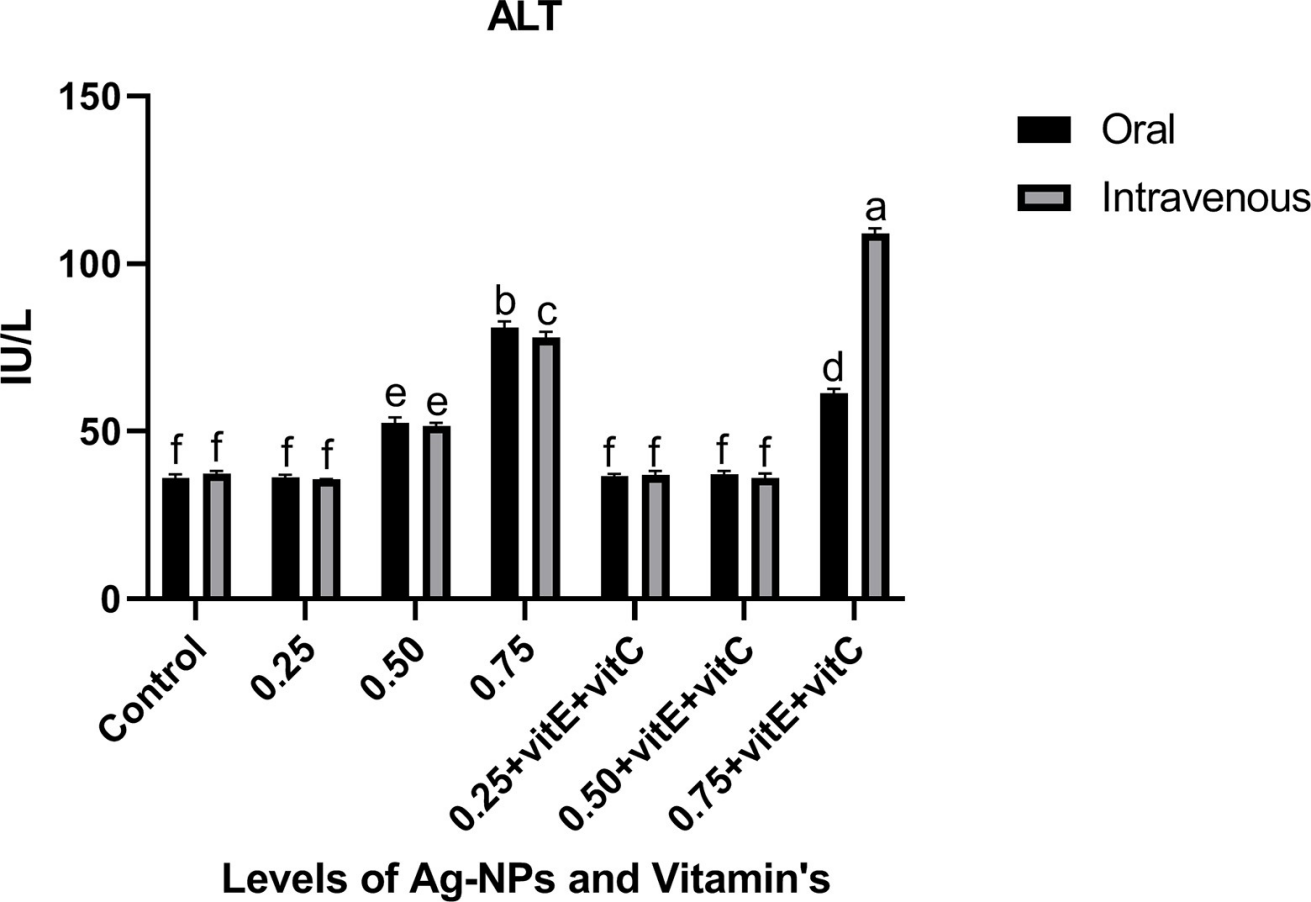
Liver function test ALT of *C*. *idella* exposed to different levels of Ag-NPs alone and Ag-NPs+vitamin E and C for 7 days period. Values represented as means ±SD (n = 3). The different superscripts show significant differences (P<0.05).

**Fig 2:**
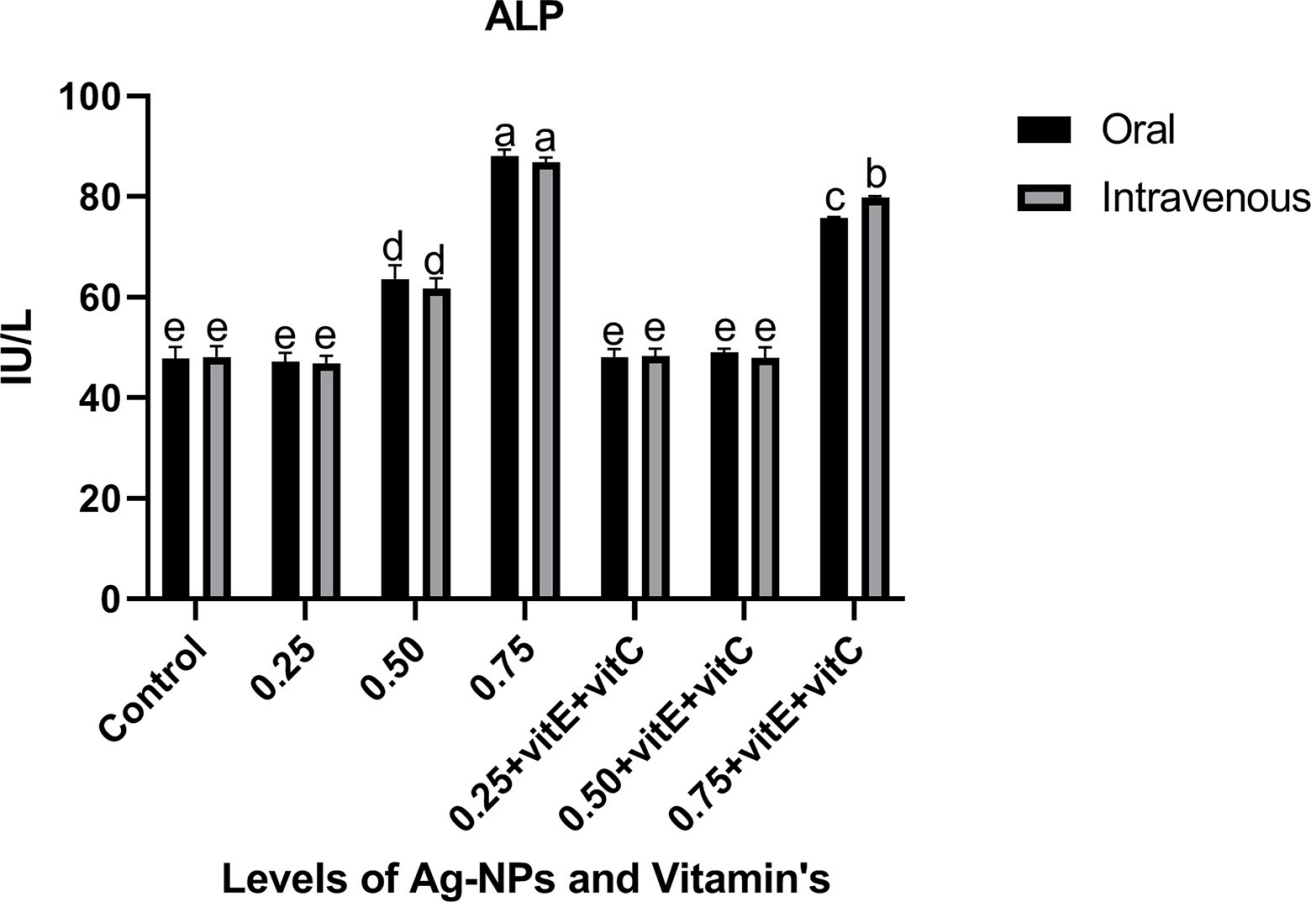
Liver function tests AST of *C*. *idella* exposed to different levels of Ag-NPs alone and Ag-NPs+vitamin E and C for 7 days period. Values represented as means ±SD (n = 3). The different superscripts show significant differences (P<0.05).

**Fig 3:**
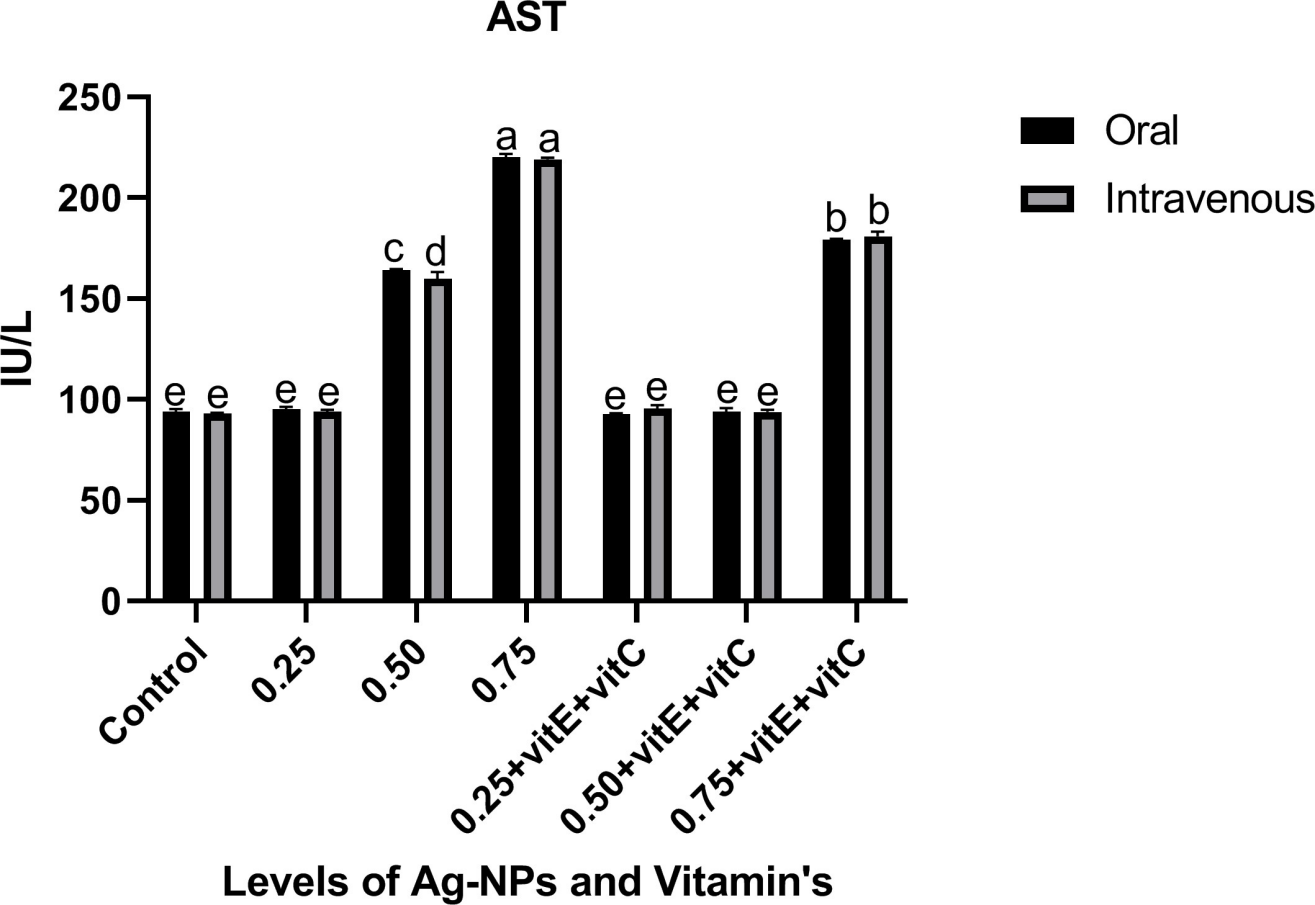
Liver function tests ALP of *C*. *idella* exposed to different levels of Ag-NPs alone and Ag-NPs+vitamin E and C for 7 days period. Values represented as means ±SD (n = 3). The different superscripts show significant differences (P<0.05).

Liver function tests (Figs 1–3) represented the significantly (P<0.05) highest levels of ALT, ALP, and AST at 0.75mg/L Ag-NPs alone and 0.75mg/L Ag-NPs with vitamin E and C groups, while all other groups showed the least values. ALT, ALP, and AST at 0.25mg/L Ag-NPs alone, 0.50mg/L Ag-NPs with vitamin E and C, and control showed non-significant (P>0.05) difference when compared. When 0.75mg/L Ag-NPs with vitamin E and C were administered intravenously rather than orally, the overall response of ALT and ALP was observed to be high. Similarly, there was a significant (P<0.05) difference in ALT levels at 0.75mg/L Ag-NPs alone between oral and intravenous mode. ALP level was significantly (P<0.05) higher at 0.75mg/L Ag-NPs alone when compared to others, and there was also a significant (P<0.05) difference in ALP levels between oral and intravenous administration at 0.75mg/L NPs with vitamin E and C. AST also showed significant (P<0.05) high level at 0.75mg/L NPs alone as compared to other groups and at 0.50mg/L NPs alone AST level showed significant (P<0.05) difference between oral and intravenous mode. However, when vitamin E and C were supplemented with Ag-NPs, it significantly (P<0.05) decreased the ALT, ALP, and AST levels, except at 0.75 mg/L of Ag-NPs.

**Fig 4:**
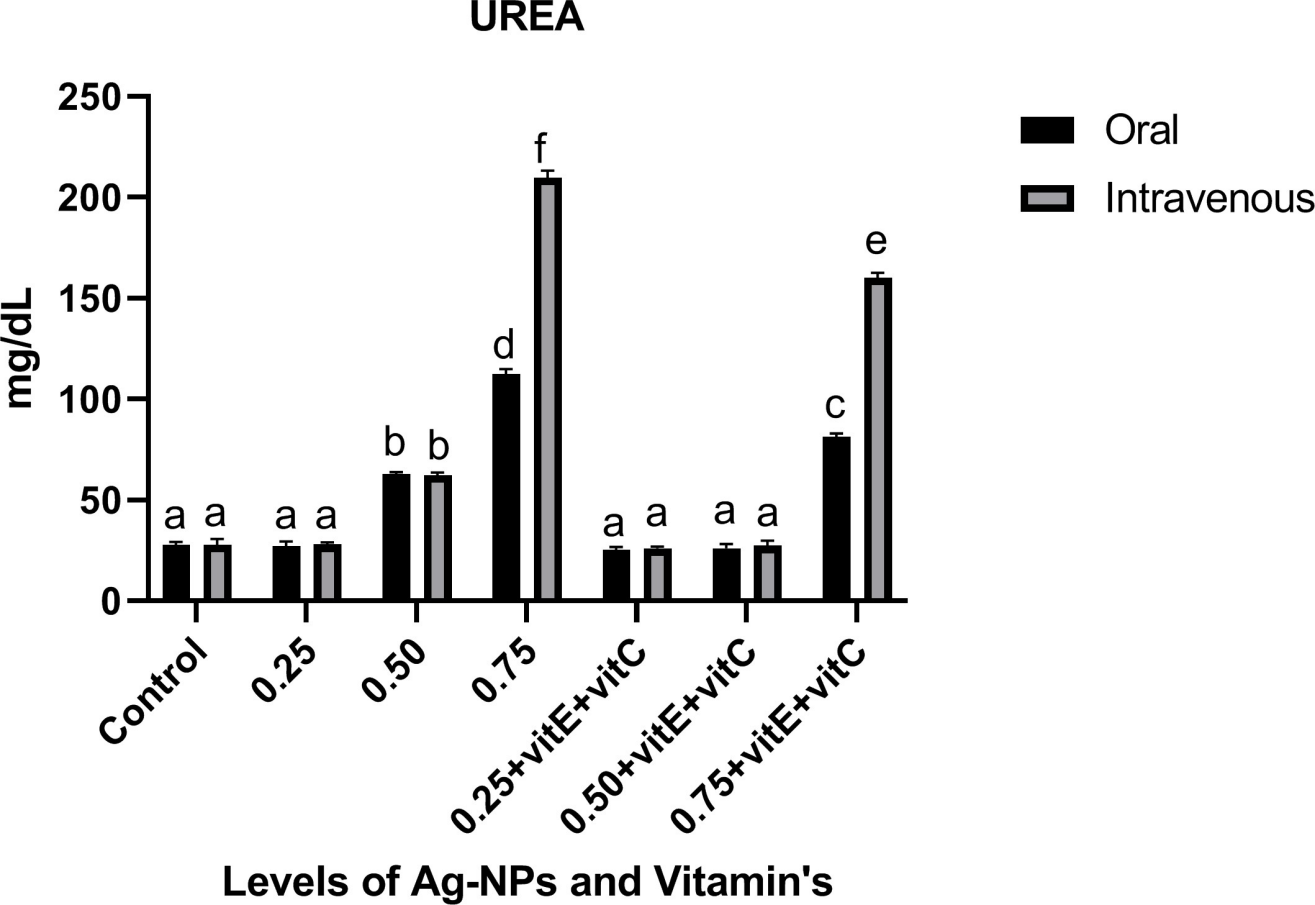
Renal function test urea of *C*. *idella* exposed to different levels of Ag-NPs alone and Ag-NPs+vitamin E and C for 7 days period. Values represented as means ±SD (n = 3). The different superscripts show significant differences (P<0.05).

**Fig 5:**
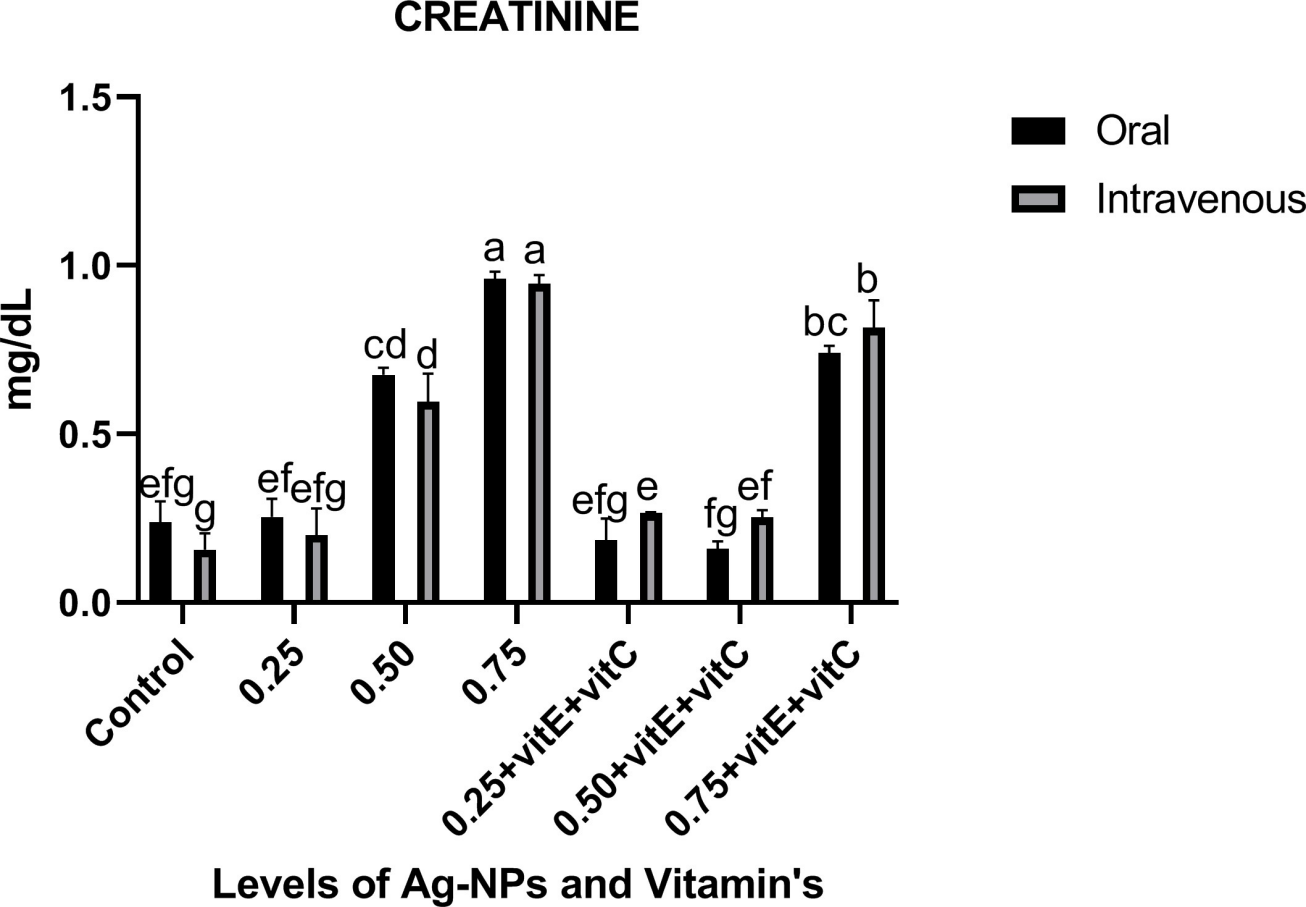
Renal function test creatinine of *C*. *idella* exposed to different levels of Ag-NPs alone and Ag-NPs+vitamin E and C for 7 days period. Values represented as means ±SD (n = 3). The different superscripts show significant differences (P<0.05).

Renal function tests (Figs 4 and 5) showed that at 0.50 and 0.75mg/L Ag-NPs alone and 0.75mg/L NPs with vitamin E and C, urea and creatinine levels were significantly (P<0.05) high as compared to control and other treatments. Urea level at 0.75mg/L NPs alone was significantly (P<0.05) higher as compared to others but also showed a significant (P<0.05) increase in urea level in intravenous mode as compared to oral mode at the same dose level (0.75mg/L NPs). Similar trend of urea level was observed at 0.75mg/L NPs with vitamin E and C, significantly (P<0.05) high in intravenous and low in oral mode. In creatinine dose of 0.75mg/L NPs showed significant (P<0.05) increase as compared to others. As vitamin E and C were ingested with NPs, urea and creatinine levels significantly (P<0.05) decreased, except at 0.75mg/L NPs + vitamin E and C.

**Fig 6:**
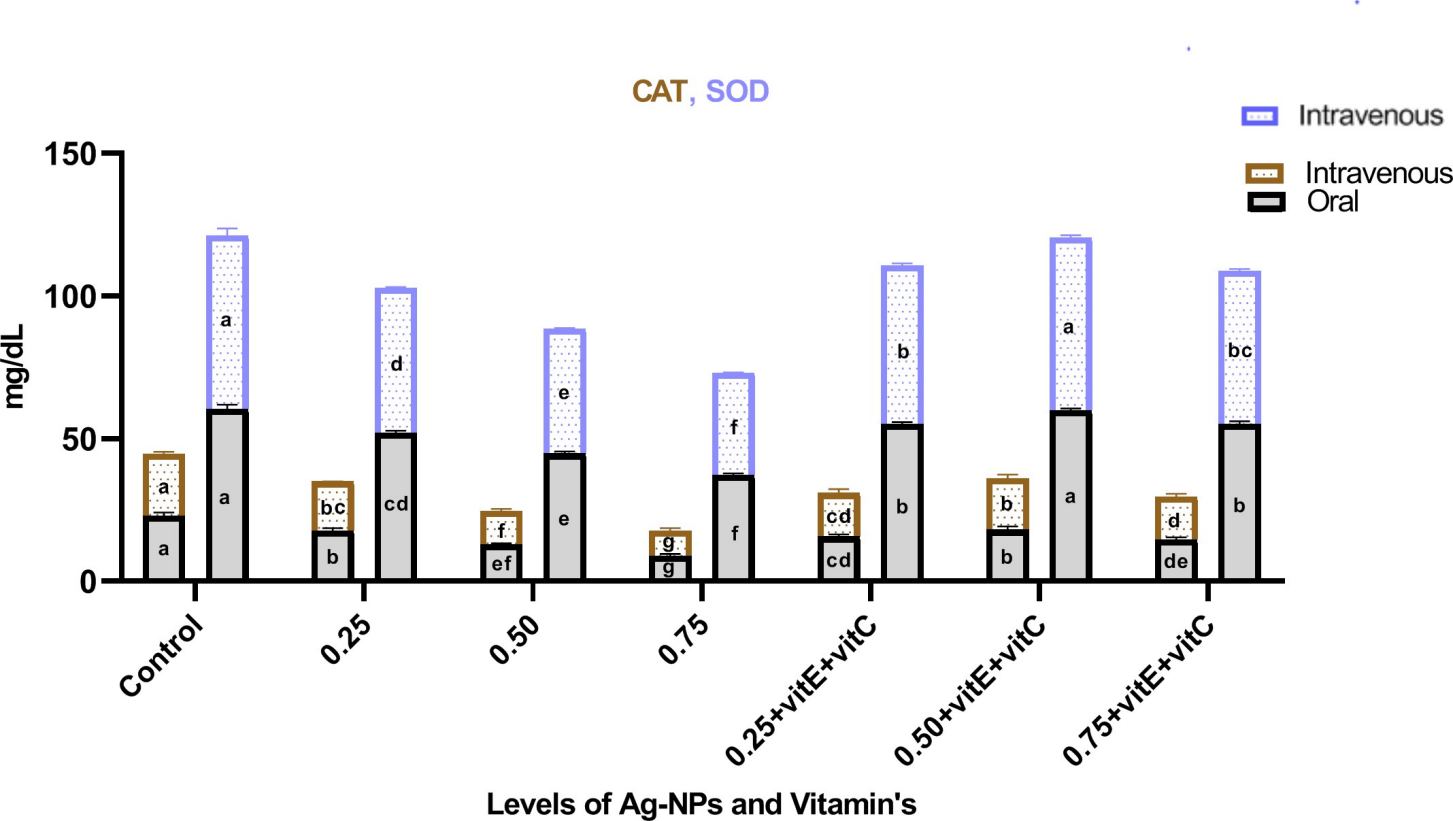
Antioxidant enzymes activity (CAT & SOD) of *C*. *idella* exposed to different levels of Ag-NPs alone and Ag-NPs+vitamin E and C for 7 days period. Values represented as means ± SD (n = 3). The different superscripts show significant differences (P<0.05).

Antioxidant enzyme activity (Fig 6) showed that as the ingestion of NPs increased from 0.25, 0.50, and 0.75 mg/L NPs, CAT and SOD enzyme activity also decreased significantly (P<0.05) as compared to control. But when the same levels of NPs (0.25, 0.50, and 0.75mg/L) were combined with 0.25, 0.50, and 0.75mg/L of vitamin E and C, CAT and SOD enzyme activity significantly (P<0.05) increased as compared to the ingestion of NPs alone. In CAT enzyme activity, ingestion of vitamin E and C causes an increased level but still a significant (P<0.05) difference with respect to control. CAT activity at dose levels of 0.25 and 0.75 mg/L NPs combined with vitamins was the same and the difference was non-significant (P>0.05) but significantly (P<0.05) high at 0.50 mg/L NPs when combined with vitamins. Similarly, SOD activity was significantly (P<0.05) lower with respect to control when NPs were combined with vitamins at levels of 0.50 and 0.75mg/L, but showed a non-significant (P>0.05) difference with control when ingested with 0.50mg/L PNs plus vitamin E and C.

**Fig 7:**
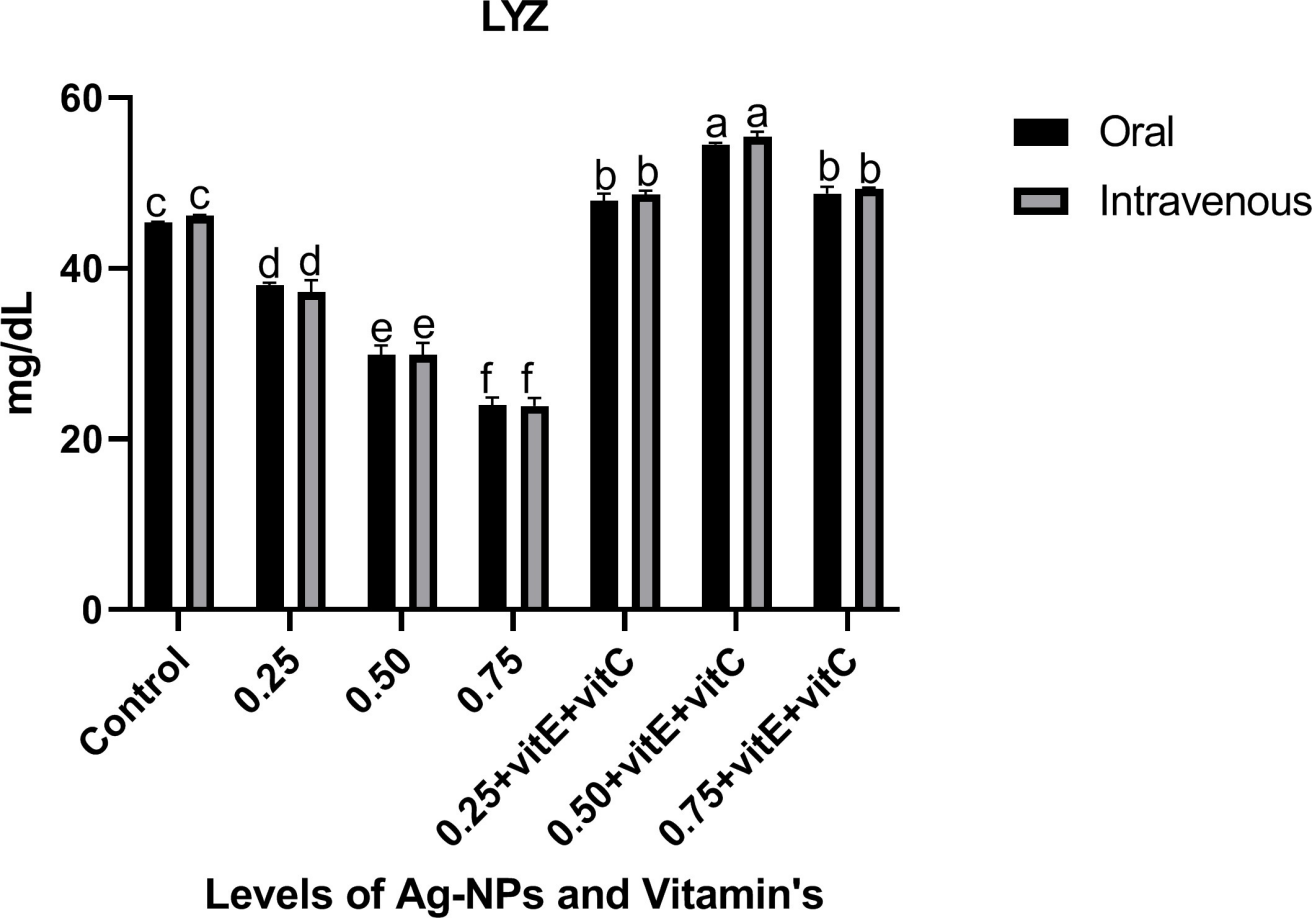
Innate immunological biomarkers (LYZ) of *C*. *idella* exposed to different levels of Ag-NPs alone and Ag-NPs+vitamin E and C for 7 days period. Values represented as means ±SD (n = 3). The different superscripts show significant differences (P<0.05).

**Fig 8:**
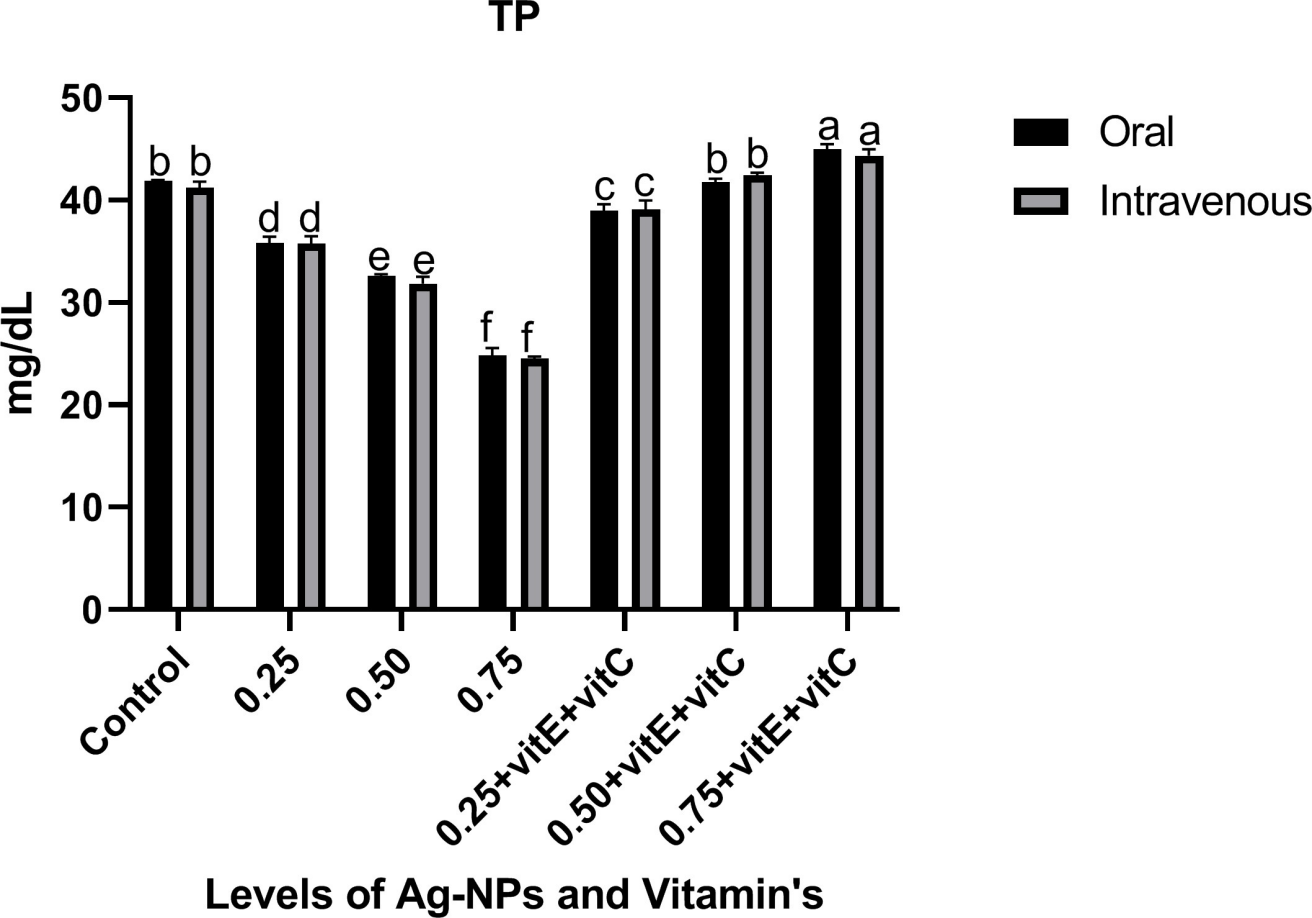
Innate immunological biomarkers (TP) of *C*. *idella* exposed to different levels of Ag-NPs alone and Ag-NPs+vitamin E and C for 7 days period. Values represented as means ±SD (n = 3). The different superscripts show significant differences (P<0.05).

**Fig 9:**
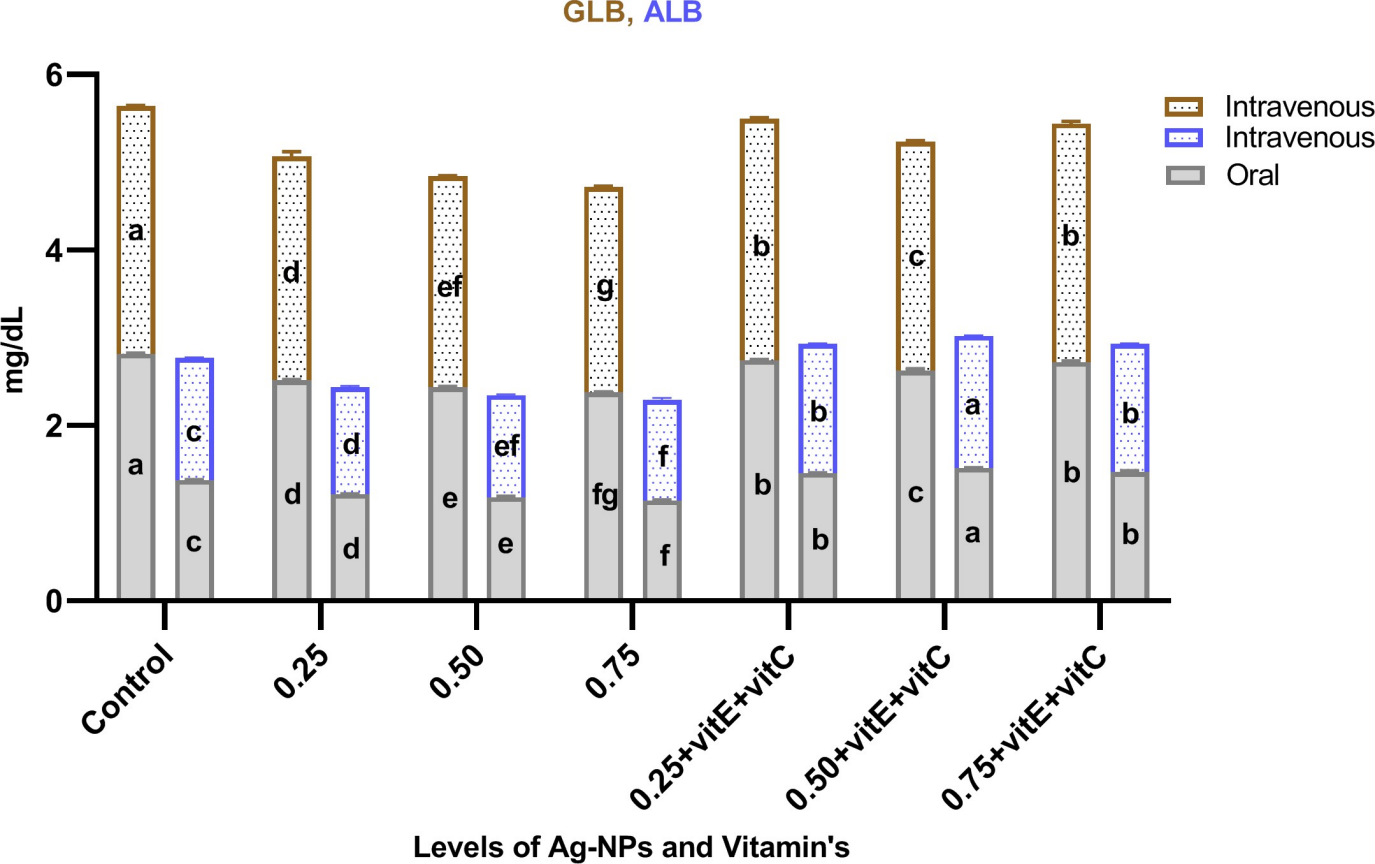
Innate immunological biomarkers (ALB, GLB) of *C*. *idella* exposed to different levels of Ag-NPs alone and Ag-NPs+vitamin E and C for 7 days period. Values represented as means ±SD (n = 3). The different superscripts show significant differences (P<0.05).

Innate immunological biomarkers like LYZ, TP, ALB, and GLB (Figs 7–9). showed significant (P<0.05) decreased activity as the dose level of NPs increased from 0.25, 0.50, and 0.75mg/L with respect to control, and the activity of these biomarkers significantly increased when ingestion of NPs was combined with vitamin E and C. LYZ and ALB activity was significantly (P<0.05) high when given with NPs plus vitamins with respect to control and significantly (P<0.05) highest activity at a dose level of 0.50mg/L NPs plus vitamins as compared to others, but there was a non-significant (P>0.05) difference at 0.25 and 0.75mg/L NPs with combined vitamins. Similarly TP activity significantly (P<0.05) increased when given with NPs plus vitamins and significantly (P<0.05) highest activity at dose level of 0.75mg/L NPs plus vitamins as compared to others. TP activity was significantly (P<0.05) lower at 0.25 mg/L NPs + vitamin E and C compared to 0.50 mg/L NPs + vitamin E and C and control. At 0.25, 0.50 and 0.75mg/L NPs + vitamins, GLB activity was significantly (P<0.05) lower as compared to control, but at 0.25 and 0.75mg/L NPs + vitamins GLB activity was significantly (P<0.05) high as compared to the 0.50mg/L NPs + vitamins dose level and non-significant (P>0.05) among them.

**Fig 10:**
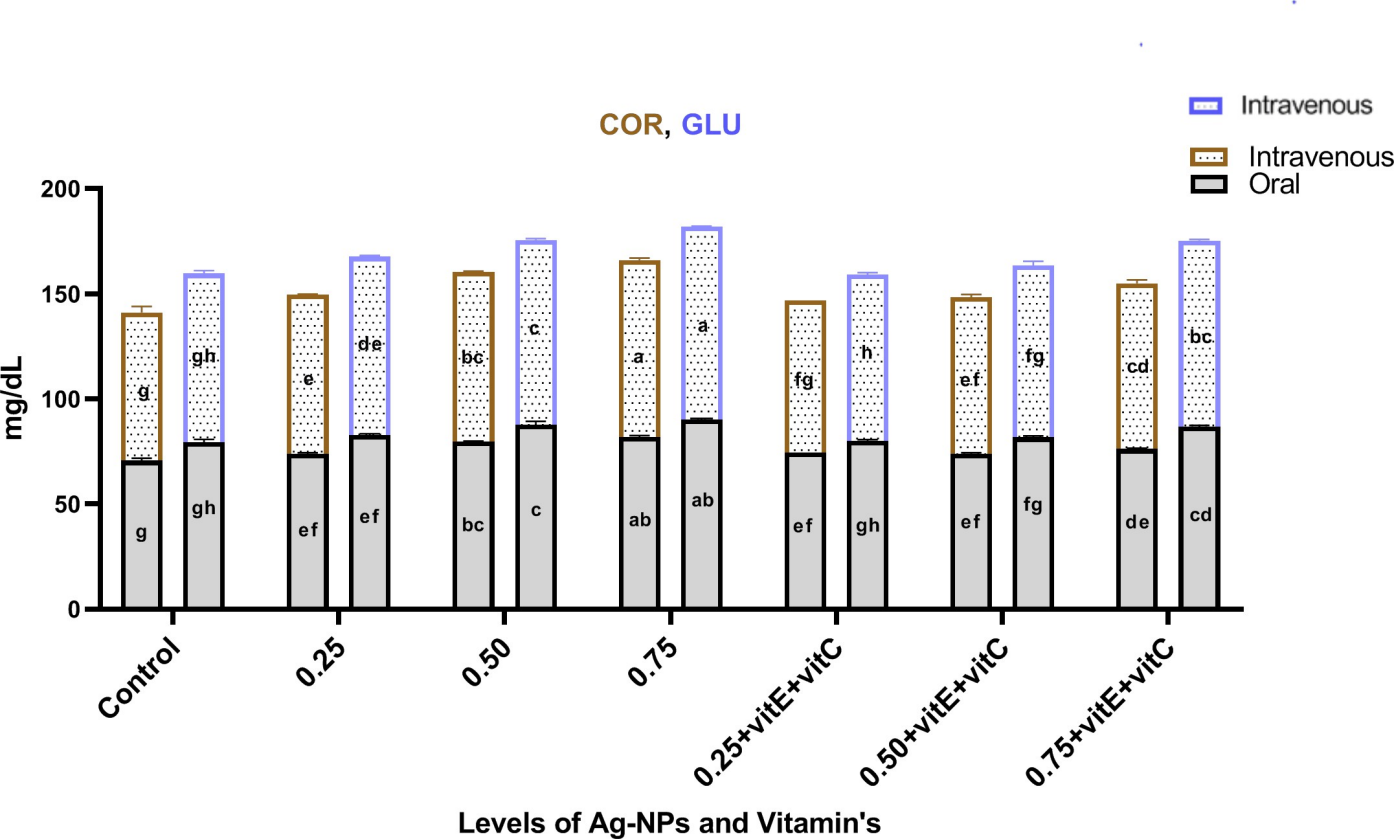
Serum stress biomarker COR & GLU of *C*. *idella* exposed to different levels of Ag-NPs alone and Ag-NPs+vitamin E and C for 7 days period. Values represented as means ±SD (n = 3). The different superscripts show significant differences (P<0.05).

**Fig 11:**
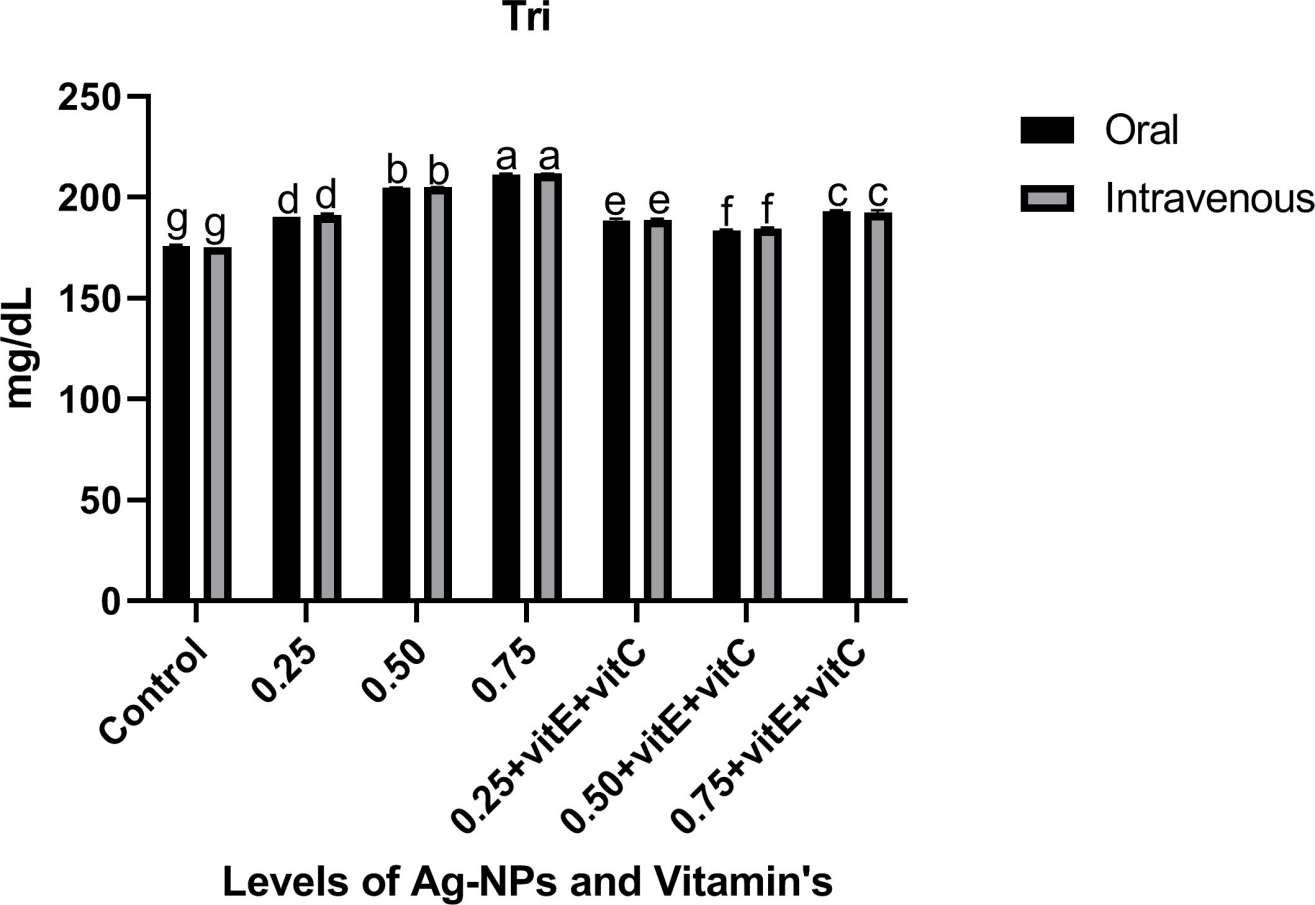
Serum stress biomarker Tri. of *C*. *idella* exposed to different levels of Ag-NPs alone and Ag-NPs+vitamin E and C for 7 days period. Values represented as means ±SD (n = 3). The different superscripts show significant differences (P<0.05).

**Fig 12:**
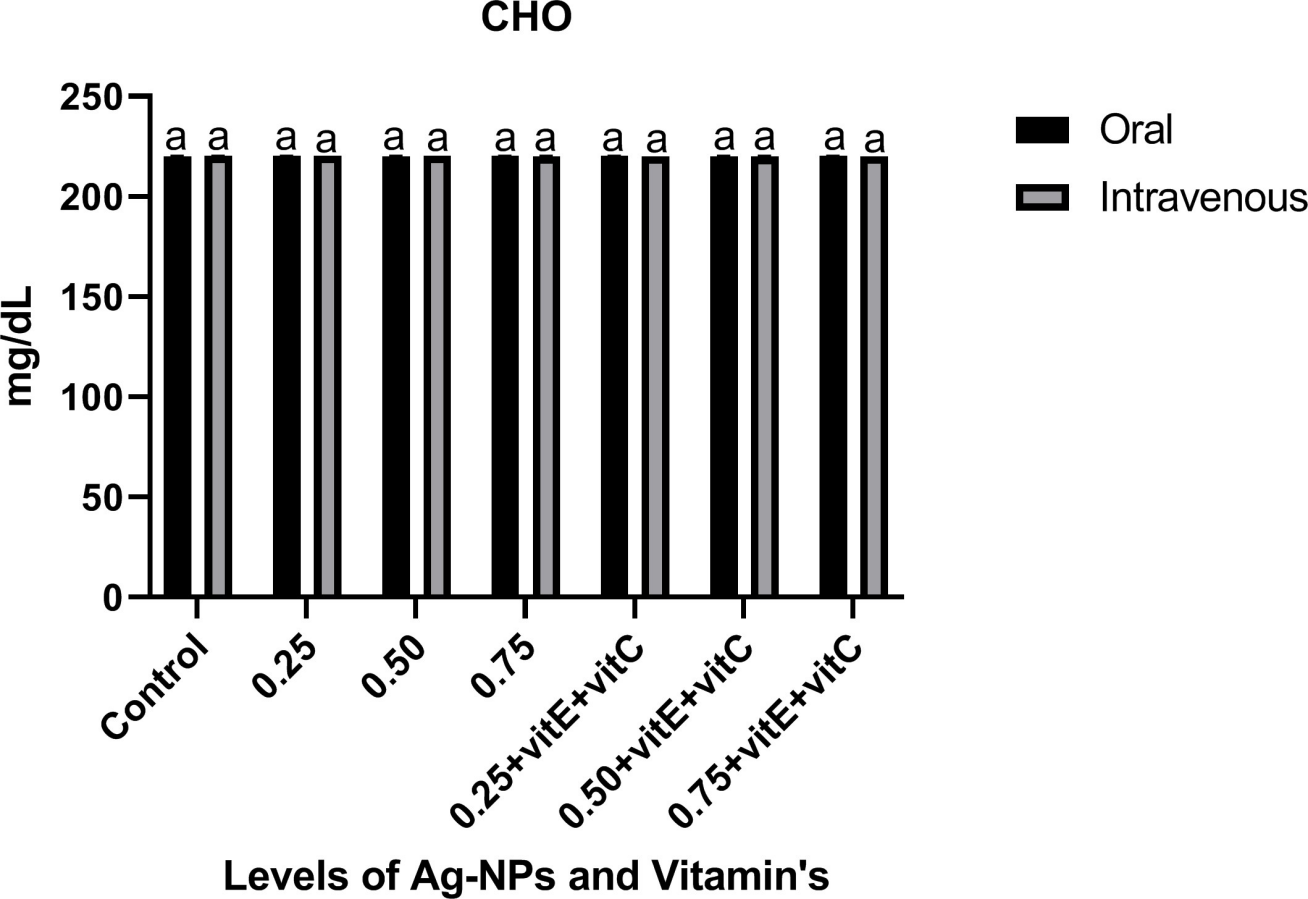
Serum stress biomarker CHO of *C*. *idella* exposed to different levels of Ag-NPs alone and Ag-NPs+vitamin E and C for 7 days period. Values represented as means ±SD (n = 3). The different superscripts show significant differences (P<0.05).

Serum stress biomarkers like triglycerides (Tri), cortisol (COR) and glucose (GLU) (Figs 10–12) showed a significant (P<0.05) increase as the dose of NPs increased from 0.25 to 0.75 mg/L alone when compared with control. When Ag-NPs were administered with vitamin E and C, these biomarkers showed a significant (P<0.05) low level at 0.75mg/L given alone in Tri, COR, and GLU. As the level of Ag-NPs increased, the effect of vitamin E and C significantly (P<0.05) decreased as compared to other treatments. Cholesterol (CHO) showed a non-significant (P>0.05) effect of NPs alone and with vitamin E and C when compared with control.

## 4. Discussion

To evaluate the status of fish health and immunity, hematological parameters are considered the best indicators for determination of toxicological and feed additive effects [2]. In the current study, we determined the effects of Ag-NPs alone and with vitamin E and C containing different dose levels through oral and intravenous mode. At dose levels of 0.50 and 0.75mg/L Ag-NPs alone, RBC, HGB, and HCT levels decreased, while WBC and NEUT levels increased significantly as compared to control, and at 0.25mg/L Ag-NPs showed a non-significant difference as compared to control, but MCV, MCH, MCHC, LYM and MONO showed non-significant effect of Ag-NPs (Table 2). Similarly, [3] reported a significant decrease in RBC and HCT and an increase in WBC and NEUT at dose levels of 25% and 50% Ag-NPs through water exposure, but non-significant difference with control at a low concentration of 12.5%, which is consistent with our findings. Ag-NPs have an adverse effect on RBC lysis because they have polyunsaturated fatty acids in their cell membrane and cause peroxidation due to toxicity [37], disrupt RBC formation [38], and cause morphological changes [39]. Another study showed a significant decrease in RBC, HCT, and Hb when treated with Ag-NPs orally in *L*.*rohita* [5]. In our study, WBC levels increased and then decreased at low and high concentrations of Ag-NPs alone and with vitamin E and C. Similar results were reported [38, 40, 41] when metals, Ag-NPs and pollutants were exposed to fish. The concentration of WBC increased with the ingestion of NPs because they act as regulators to enhance the immune system in fish in the presence of toxicity in the body [42]. Ag-NPs + vitamin E and C ingestion at the level of 0.25 and 0.50mg/L showed RBC, HGB and HCT parameters significantly increased and WBC and NEUT significantly decreased as compared to Ag-NPs alone groups. Vitamin E and C have strong antioxidant properties that protect the hemolysis of HB and RBC and cause enhancement in their level. The reason for the decrease in WBC and NEUT is the positive effects of vitamin E and C on reducing the oxidative damage caused by NPs [43]. At 0.75mg/L NPs + vitamin E and C, hematological parameters showed a significantly low level and WBC and NEUT showed a significantly high level with respect to other vitamin supplemented groups. Same results were observed when TiO2-NPs supplemented with vitamin C [44]. The high concentration of TiO2-NPs cancelled the protective effect of vitamin E and C on hematology and shifted the curative effect of vitamin E and C on other organs of the body like the gills, kidney, and liver.

**Table 2:** Interaction of dose levels and dose route i.e. Oral and Intravenous effect of Ag-NPs alone and Ag-NPs + V.E + V.C on hematology of *C*. *idella*.

Dose Levels	Dose Route	Parameters									
		RBC (10^6^/μl)	WBC (10^3^/μl)	HGB (g/dl)	HCT (%)	MCV (nm^3^)	MCH (pg/cell)	MCHC (g/dl)	LYM (%)	MONO (%)	NEUT (%)
**A**	**Oral**	2.09±0.01	171.04±0.46	9.15±0.03	35.32±0.75	168.10±1.25	168.10±1.25	31.28±0.81	82.96±1.00	2.50±0.08	21.37±1.19
	**Intra**	2.09±0.01	172.44±1.54	9.15±0.02	36.01±0.31	168.26±2.39	168.26±2.39	30.94±0.67	82.00±1.35	2.38±0.05	20.76±0.91
**B**	**Oral**	2.08±0.01	172.99±0.27	9.18±0.02	35.27±0.83	168.99±1.61	168.99±1.61	30.48±0.32	82.71±1.38	2.34±0.02	21.02±1.53
	**Intra**	2.08±0.01	172.42±1.69	9.16±0.02	35.28±0.89	167.27±1.52	167.94±1.28	30.92±0.49	82.21±1.32	2.40±0.08	21.75±1.03
**C**	**Oral**	1.94±0.12	185.73±3.95	8.29±0.01	30.14±0.46	166.10±0.50	167.43±1.34	31.56±0.59	81.60±1.80	2.35±0.02	26.48±0.83
	**Intra**	1.94±0.11	186.23±0.87	8.28±0.02	29.80±0.66	166.46±1.00	167.79±0.98	30.78±0.50	83.26±1.55	2.35±0.00	26.79±1.02
**D**	**Oral**	1.60±0.02	223.02±4.23	6.85±0.19	23.06±0.51	167.44±1.95	168.11±1.34	31.09±1.24	81.98±0.43	2.39±0.07	31.80±1.19
	**Intra**	1.57±0.02	223.60±1.20	6.74±0.13	23.62±0.70	166.10±0.62	167.77±1.09	30.27±1.04	81.45±0.33	2.40±0.06	32.00±1.19
**E**	**Oral**	1.92±0.31	172.04±1.13	9.16±0.03	35.98±0.78	166.04±1.20	167.71±0.57	30.47±0.60	83.81±1.29	2.48±0.07	21.50±1.21
	**Intra**	1.93±0.27	172.99±1.56	9.14±0.01	35.18±0.94	166.78±0.78	167.11±0.39	30.20±0.53	82.53±1.10	2.40±0.15	21.03±0.34
**F**	**Oral**	2.09±0.00	172.06±2.06	9.16±0.04	36.37±0.17	166.75±1.78	168.75±1.16	30.70±0.69	82.95±1.11	2.44±0.10	21.39±0.51
	**Intra**	2.07±0.01	172.74±1.25	9.14±0.02	35.45±0.86	167.77±2.16	168.10±1.59	30.35±0.48	83.30±1.13	2.43±0.11	21.13±0.78
**G**	**Oral**	1.78±0.02	204.54±0.08	7.99±0.04	26.83±0.46	166.31±1.57	166.97±0.83	30.69±1.05	82.54±1.96	2.34±0.08	28.17±0.89
	**Intra**	1.77±0.02	201.34±1.89	7.99±0.02	26.80±0.29	168.04±1.31	168.04±1.31	30.76±1.07	82.56±1.07	2.35±0.03	28.28±0.84
Dose*Route (P-value)		1.000	0.48	0.79	0.29	0.43	0.83	0.84	0.84	0.49	0.91

Data are presented as mean ± SD. Data were analyzed through two-way ANOVA besides Duncan comparisons. Data with different superscript letters (a, b, c, d, e and f) in the same column mean significant differences among experimental groups (P<0.05).

In the current research, liver function tests showed that at 0.50, 0.75mg/L Ag-NPs alone, and 0.75mg/L Ag-NPs + vitamin E and C, the levels of ALT, ALP, and AST were considerably high. Similarly [45] found that NPs toxicity damages the liver and raises the ALT, AST, and ALP enzymatic activity, similar to our research. A high concentration of Ag-NPs with vitamins E and C, according to [25], totally alleviate liver damage. Ag-NPs have the ability to harm fish cells and produce ROS that are poisonous and induce oxidative stress in the fish’s body [2, 46]. Ag-NPs infiltrate the cell membrane by diffusion and produce reactive oxygen species (ROS), which damage the mitochondria, proteins, DNA, and acids in hepatocytes [4, 47]. Ag-NP overdose caused hepatocyte destruction, which resulted in significantly elevated levels of ALT, ALP, and AST in the current investigation. Any toxicity or injury to the hepatic cells results in a change in the activity of these enzymes, which include AST, ALT, and ALP in the hepatic cell membrane [48, 49]. The activity of ALP, AST, and ALT is markedly reduced when vitamin E and vitamin C are given together with NPs. [50] also noted that vitamin E had a curative effect when combined with ZnO-NPs and that ALP, AST, and ALT activity had decreased. Liver enzyme activity is decreased by vitamins E and C [51]. By lowering liver enzyme activity, [25, 52] identified the antioxidant effect of vitamin C and vitamin E against ZnO-NP in fish.

Renal function tests demonstrated that at 0.50, 0.75mg/L Ag-NPs alone and 0.75mg/L Ag-NPs with vitamin E and C, urea and creatinine levels were significantly high as compared to control and other treatments. As vitamin E and C were ingested with Ag-NPs, urea and creatinine levels significantly decreased, except at 0.75mg/L Ag-NPs + vitamin E and C. The decrease in urea and creatinine may be due to renal damage, but it is also associated with liver and gill damage because of Ag-NPs. The exchange of these two parameters could not take place properly between water and blood in gills, so it causes the elevation of these parameters [25]. Similar to our results, a decrease in urea and creatinine against ZnO-NPs by supplementing vitamin E and C in fish [25].

The cytotoxicity of Ag-NPs leads to the production of reactive oxygen species (ROS), which in turn leads to oxidative stress in fish [53]. Therefore, ROS controls lipid peroxidation, membrane permeability shifts, oxidative damage, protein carobonylation, and DNA damage [54]. When Ag-NPs release metal ions, SOD reacts with them. Antioxidant enzymes like SOD and CAT help neutralise free radicals from metal ions, and CAT also converts H2O2 to water [55]. In this work, the ingestion of Ag-NPs led to a shift in CAT and SOD levels and an inhibition of their activities. When Ag-NPs were combined with vitamins E and C, the enzyme activity of the CAT and SOD groups recovered to pre-Ag-NPs levels. Consistent findings were also reported by [46, 54]. Another work by [56] shown that exposure to Ag-NPs reduces CAT activity, which in turn impacts the inhibition of SOD and CAT activity. Damage to fish organs from an excess of reactive oxygen species and the breakdown of antioxidant defence systems result from this. Vitamin E’s therapeutic effects against ZnO-NPs in *Oreochromis niloticus* were also shown by [52], who also revealed the same level of antioxidant enzyme activity as in the control group. In addition to boosting antioxidant defences, vitamin C has been shown to boost SOD and CAT activity on its own [57]. Vitamin E and C scavenging antioxidant activity was also observed by [2, 52].

TP, ALB, and GLB showed significantly decreased activity with the ingestion of Ag-NPs, and the activity of these biomarkers significantly increased when ingestion of Ag-NPs was combined with vitamin E and C. Innate immunological biomarkers like TP, ALB, and GLB are very important serum proteins that liver tissues produce and store. These biomarkers decreased in concentration, weakened the immune system, and reduced feed intake in cases of liver damage caused by toxicology [29, 58]. According to [38], Ag-NPs induce toxicity that causes the usage of these proteins due to stress and also damage kidney tissues, intestine damage and disrupt protein synthesis due to disorder in amino acid absorption. In line with our study, [59] reported that Ag-NPs reduce the TP, ALB, and GLB in Zebra fish. Similarly, [60] observed increased activity of total protein, albumin, and globulin when fish were administered with vitamin E. Lysozyme (LYZ) activity with the ingestion of NPs alone decreased and significantly increased when NPs were combined with vitamin E and C. In fish immunity, LYZ is an essential part of innate immunity and leukocytes produce and secrete it, which has extraordinary antimicrobial enzyme activity [61]. The liver produces immune globulin proteins that aid LYZ in phagocytosis and increase leukocyte activity [62]. In line with our findings, [63] demonstrated that Ag-NPs bind to these proteins, which then bind to LYZ to form the protein corona described in previous studies, depleting immune cells and causing significant oxidative damage. That’s why, in our study, LYZ activity impaired, increased the fish sensitivity towards disease and pathogens, and destabilized the immune system. In relation to our results, [64] reported that vitamin E and C significantly increase the total protein, LYZ, albumin, and globulin. Other studies also mentioned that vitamin C alone has strong antioxidant effects and reduces the toxic effects of NPs and increases the levels of TP, ALB, GLB, and LYZ in fish [25] and also has the ability to enhance leukocyte activity [65], lysozyme activity [66] and serum GLB and TP [67] to make the immune system strong.

GLU, Tri, and COR are three crucial biomarkers for assessing the severity of stress caused by disease or poisoning in fish [68]. In the current investigation, the consumption of Ag-NPs resulted in a considerable increase in Tri, GLU, and COR. Our findings are consistent with those of previous toxicity investigations of Ag-NPs [2, 69]. Adrenal cortex cells, which secrete cortisol and control glucose output from the liver through glycogenolysis and gluconeogenesis, are affected by Ag-NPs [70]. When under stress, the body responds by increasing GLU levels in the blood as a means of mitigating the negative effects of the situation. Cortisol levels that are too high are associated with liver damage and immune system suppression [38, 71]. Fish exposed to Ag-NPs have greater blood triglyceride levels, which gives the fish more energy to deal with the toxicological stress [72]. While Ag-NPs alone and in conjunction with vitamin E and C had no influence on cholesterol levels, similar to the findings of [3]. Our results demonstrated a considerable reduction in these indicators after ingestion of Ag-NPs with vitamins E and C. Vitamin E has a scavenging action and can restore cortisol, glucose, and lipid levels, according to research by [50, 52].

## 5. Conclusion

The current research shown that grass carp (*C*. *idella*) are protected against Ag-NPs toxicity by vitamin E and C as a powerful antioxidant. Restoring biomarker levels to those seen in the control group demonstrates protection. Except for the very lethal 0.75mg/L dosage of Ag-NPs, grass carp seem to be totally protected by vitamins E and C.
